# Harnessing Food Product Reviews for Personalizing Sweetness Levels

**DOI:** 10.3390/foods11131872

**Published:** 2022-06-24

**Authors:** Kim Asseo, Masha Y. Niv

**Affiliations:** The Institute of Biochemistry, Food Science and Nutrition, The Robert H. Smith Faculty of Agriculture, Food, and Environment, The Hebrew University of Jerusalem, Rehovot 76100, Israel; kim.asseo@mail.huji.ac.il

**Keywords:** taste, sweetness, NLP, sensory, hedonic, nutrition, health

## Abstract

Sweet taste is innately appealing, ensuring that mammals are attracted to the sweetness of mother’s milk and other sources of carbohydrates and calories. In the modern world, the availability of sugars and sweeteners and the eagerness of the food industry to maximize palatability, result in an abundance of sweet food products, which poses a major health challenge. The aim of the current study is to analyze sweetness levels, liking, and ingredients of online reviews of food products, in order to obtain insights into sensory nutrition and to identify new opportunities for reconciling the palatability–healthiness tension. We collected over 200,000 reviews of ~30,000 products on Amazon dated from 2002 to 2012 and ~350,000 reviews of ~2400 products on iHerb from 2006 to 2021. The reviews were classified and analyzed using manual curation, natural language processing, and machine learning. In total, ~32,000 (Amazon) and ~29,000 (iHerb) of these reviews mention sweetness, with 2200 and 4600 reviews referring to the purchased products as oversweet. Oversweet reviews were dispersed among consumers. Products that included sucralose had more oversweet reviews than average. 26 products had at least 50 reviews for which at least 10% were oversweet. For these products, the average liking by consumers reporting oversweetness was significantly lower (by 0.9 stars on average on a 1 to 5 stars scale) than by the rest of the consumers. In summary, oversweetness appears in 7–16% of the sweetness-related reviews and is less liked, which suggests an opportunity for customized products with reduced sweetness. These products will be simultaneously healthier and tastier for a substantial subgroup of customers and will benefit the manufacturer by expanding the products’ target audience. Analysis of consumers’ reviews of marketed food products offers new ways to obtain informative sensory data.

## 1. Introduction

Humans have an innate preference for sweet foods and have been shown to eat highly sweet foods since at least the stone age [1,2,3]. This attraction helps with the acceptance of breastmilk in infancy and later with fruits and other sources of nutrition during periods of growth [4]. Preference for sweetness intensity is dependent on age, gender, race and culture, individual differences, and the food or beverage itself [1,5].

Food choice does not necessarily fully align with food preference and depends on other factors, such as price and health [6]. Moreover, the desire to eat something sweet depends on social norms, the behavior of others at the table, and the satiety. While people have a suppressed appetite for sweet foods after eating something sweet or when experiencing general satiety, highly palatable and easily accessible food can cause the opposite effect and increase sweet food consumption [5]. Although sweetness preference does not correlate with obesity and type 2 diabetes [6,7,8], overconsumption of sugars does [1,9]. The availability of sugars and sweeteners and the eagerness of the food industry to maximize appeal to customers result in sweet food product abundance, posing a major health challenge [10].

Obesity is a global pandemic affecting over 650 million adults (13%), with 378 million children under 18 classified as overweight or obese as of 2016 (World Health Organization, 2020). The World Health Organization (WHO) and the 2015–2020 Dietary Guidelines for Americans (DGA) recommend an intake of less than 10% of total calories from added sugars. Moreover, the 2020–2025 DGA committee was considering lowering the limit to 6%, although the official recommendation stayed at 10% [11,12,13]. Higher added sugar intake not only increases the risk of certain diseases but also correlates with poorer diet quality [1].

While there was some decrease in the consumption of sweet beverages and tabletop sweeteners from 2001 to 2018 in the U.S., consumption of sugars and other sweeteners from general foods did not decrease [14], indicating the need to find better ways to lower sugar intake, especially from sweet food products.

Approximately 72% of Americans were trying to limit or avoid sugars in 2021 (80% in 2019), and 23% prefer using low- and non-caloric sweeteners [15], though there is accumulating evidence of the deleterious effects of certain non-caloric sweeteners [16,17,18].

Food companies are aiming to optimize the sensory profile and liking of the products, by measuring taste perception and hedonics using multiple sensory evaluation techniques, such as descriptive sensory analysis, hedonic and preference tests [9,19]. Sweetness ratings can be achieved using various methods, and though the scores depend on the measurements, the scales and the context [9,20], optimal concentration of sucrose is mostly consistent among different test conditions [20].

Sensory tests are key for launching and monitoring the success of a food product and typically rely on 100 to 1000 participants that are expected to represent the consumers [21]. 

Recent analyses of big online data emerged as a rich additional source of information: the usability of Google searches on taste loss for monitoring COVID-19 was suggested and scrutinized [22]; social media was used to identify food trends and dietary habits; open-source media outlets and online food reviews have been used to identify unsafe food products; online recipes were used to build flavor-pairing patterns, and recommendation systems were designed for providing recipe suggestions based on ingredients or health requirements [23].

A relatively large (~2300 participants) study examined the relationship between liking and sensory perception of foods and found that degree of liking grew in parallel with sweetness responsiveness [24]. Large studies based on free text and reviews typically rely on natural language processing tools. 

### 1.1. Theoretical Background on Using Natural Language Processing

Natural language processing (NLP) applies a combination of linguistics, computer science, and artificial intelligence approaches to human language. The goal is to provide computerized “understanding” of the contents of documents, including the contextual nuances of the language within them.

Tools and data processing methods in NLP are task dependent. Below are two examples of data pre-processing and some adjustments considerations:

Stemming is usually applied in the pre-processing stage when analyzing text to converge word with the same root meaning that appear in different forms, usually by removing the suffixes [25]. For example, the words “cook”, “cooks”, “cooking”, and “cooked” becomes “cook”. Stemming is used to reduce the vocabulary and noise in the data and increase word frequencies. One disadvantage of using stemming is that sometimes the stem is hard to interpret (e.g., the stem of “convenient” is “conveni”). Another problem can arise when the intention and meaning of phrases depend on these suffixes. For example, the stem of “sweet”, “sweets”, and “sweetness” is "sweet", but “sweet” as an adjective is more meaningful than “sweets” as a noun when analyzing sentiment in text about food.

An additional pre-processing stage is the removal of stop words and low frequency words [25]. The list of words to be filtered out depends on the context of the analysis. For example, an analysis focused on sentiment will potentially avoid removing words such as “very”, “too”, “like”, “not”, “happy”, as opposed to analysis focused on extracting names in which removing pronouns and verbs can potentially reduce the vocabulary without losing necessary information. The cut-off for low frequency words is also an important factor that depends on the application. Choosing a high cut-off will reduce dimensionality but can potentially miss rare and important terms related to the task. Rare terms are sometimes used to emphasize opinions [26] and thus might be useful in sentiment analysis tasks. 

NLP for sentiment analysis can be applied, for example, to determine hedonics from comments regarding food or for understanding personal differences [27]. For example, comments such as “This tea was absolutely delicious.”, “This chocolate was ok. I guess not too bad, but I probably won’t buy it again.”, and “This was horrible! If I could give 0 stars I would! Yuck!!” can be categorized as positive, neutral, and negative, respectively, without the need for manual tagging. 

A recent application of NLP used whisky reviews to develop a descriptive lexicon [28] where 6600 reviews were used to produce an automated whiskey lexicon as a case study for speeding up descriptive methods. A similar study used comments from Chinese e-commerce websites to build a lexicon for crab meat [29].

Using NLP in sensory analysis provides a way of analyzing a large amount of data in a short time, thus supplementing the time-consuming and costly traditional sensory techniques. Indeed, recent studies employing NLP techniques examined the helpfulness of reviews [30], the effect of sentiment on food choice and expectations [31,32], and prevalence of reviews discussing oversweetness of products [27].

### 1.2. Aim of the Current Study

We hypothesized that analysis of sweetness levels, liking, and ingredients might provide novel insights into the taste–health conflict. We analyzed the ingredients and reviews of food products sold on Amazon (www.amazon.com, last accessed on 20 November 2020) and iHerb (www.iherb.com, last accessed on 22 June 2022) to evaluate how consumers perceive and enjoy the products in a natural setting. To the best of our knowledge, this is the first big-data analysis that links food reviews to their ingredients. Importantly, the analysis provides insights on the preferred type of sweetener people use, assuming that customers buy products they want to eat. 

## 2. Methods

### 2.1. Data Curation

A dataset of reviews for products sold on Amazon was obtained from the “Amazon fine food reviews” dataset on Kaggle, an open-source data competition site (Stanford Network Analysis Project, Amazon Fine Food Reviews, www.kaggle.com, version 2, 2017, https://www.kaggle.com/snap/amazon-fine-food-reviews, accessed on 15 February 2020 [33]). The data contained 393,579 unique reviews of 74,258 products from October 1999 to October 2012. This dataset contained product IDs, customer profile IDs, text and titles, dates posted, and the ratings (on a 1 to 5 stars scale).

Product information was obtained by scraping Amazon using the product ID as the identifier. Scraping was conducted by loading the product URLs indicated by the product ID and extracting the products’ names, categories, and ingredient lists. Products with unavailable URLs were eliminated, along with products lacking an ingredient list or not listed under food products for humans. Some of the information did not match; for example, headphones appearing under the pasta category. These products were also eliminated. A possible explanation for these inconsistencies can be that the URLs were reused for newer products, while the updates to these pages were performed incorrectly. After the required eliminations, the data contained 215,975 unique reviews for 29,060 products dated from November 2002 to October 2012 (mean December 2010). The current Amazon reviews dataset holds ~216,000 reviews out of the ~394,000 reviews used by Reed et al. [27].

A dataset for iHerb products and reviews was obtained by scraping products under the ‘grocery’ category on iHerb.com using Octoparse version 8 (www.octoparse.com). The scraper went over all web pages under this category, entered the link to each product page, and extracted the necessary product information. Following that, each product page URL was used to access its review page, which was subsequently accessed to extract all its reviews. The data contained 349,612 unique reviews for 2408 products from May 2006 to November 2021 (mean January 2020).

The data contained the following for each product: an identifier, a name, an ingredient list, a category, and all reviews. Each review contained the product identifier, the customer profile URL, the text and title, and the date posted. Review ratings for some products (on a 1 to 5 stars scale) were obtained by additional scraping approximately six months after scraping for the reviews themselves. Products without reviews or ingredient lists were eliminated. 

The ingredients for all products were extracted, regular expressions were used to eliminate all filler words, unnecessary punctuation and spaces, and the remaining were established as the ingredient list. This list was used to find all the sweeteners used in these products and all possible spellings or phrasings for these sweeteners. Columns indicating the presence of each sweetener in the ingredient list of each product were added to the product information. 

### 2.2. Word Search

Word use analysis was performed based on the methods described in [27] with the addition of the following words for the taste category: flavor, flavour, saltish, tart, tangy, and biter (a common misspelling of “bitter”).

### 2.3. Sweetness Level

Phrases containing the word “sweet” were obtained similarly to the methods used by Reed et al. (2019) [27]. Duplicated reviews were removed, and all one-word environments containing the word “sweet” were extracted from the text. These environments were used to identify all possible phrases indicating over, under, and neutral sweetness levels.

Common negations of those words were added before each phrase to create negative versions of the phrases (“isn’t very sweet”), along with common misspellings (“isnt very sweet”). Moreover, we added to each of these phrases either suffixes that indicate that the phrase is not about sweetness level (e.g., “sweet tooth”, “sweetener”, “sweet leaf”) or the word “enough”, which can change the meaning of the overall phrase.

The phrases were categorized by sweetness level (oversweet, under-sweet, and neutral), and the number of reviews was tallied for each category (see List S1 in the Supplementary Information for the complete list of phrases and their classification by sweetness level). In addition, the number of occurrences of the word “sweet” that appeared in one of the previously mentioned phrases was counted.

### 2.4. Review Classification by Oversweetness

Reviews that contained an oversweet phrase were classified into either oversweet that refers to the product or not oversweet (meaning reviews that contained one of the phrases but did not refer to the oversweetness of the product). All Amazon reviews and 71% of iHerb reviews were classified manually. The manually labeled iHerb reviews were randomly divided into: 75% training set (*n* = 4192) and 25% test set (*n* = 1398) for an XGBoost classifier [34]. The features used were the 99.5% most frequent words in the iHerb reviews. The bag-of-words XGBoost model was evaluated on the training set (with cross-validation, k-fold = 10), test set, and holdout set (400 reviews randomly sampled from the unlabeled reviews and checked after applying the classifier).

Parameter tuning was performed using sklearn’s GridSearchCV from sklearn.model selection module [35] with 10 k-fold cross-validation and scoring method set to ‘f1 score’ to improve precision and recall. The rest of the iHerb reviews were classified by XGBoost.

Model construction, fitting, and parameter tuning were conducted using Python version 3.7.3 (https://www.python.org/).

### 2.5. Statistical Analysis

The tendency of individual sweeteners’ to be classified as oversweet was calculated by comparing the number of oversweet reviews out of the total reviews for products containing each sweetener to the proportion of oversweet reviews out of the total reviews for each dataset. The *p*-values for proportion comparison were computed using a two-sided proportion test with a confidence interval set to 0.95, implemented by the ‘prop.test’ function, and adjusted using the Bonferroni correction.

The *p*-values for comparing the average ratings of oversweet and not oversweet reviews were calculated by a one-sided Wilcoxon rank-sum test and adjusted for multiplicity using the Benjamini–Hochberg procedure [36]. A level of *p* < 0.05 was used to determine statistical significance.

Analyses and visualizations were conducted using the statistical software R version 4.0.2 (https://www.r-project.org/).

## 3. Results

**Figure 1:**
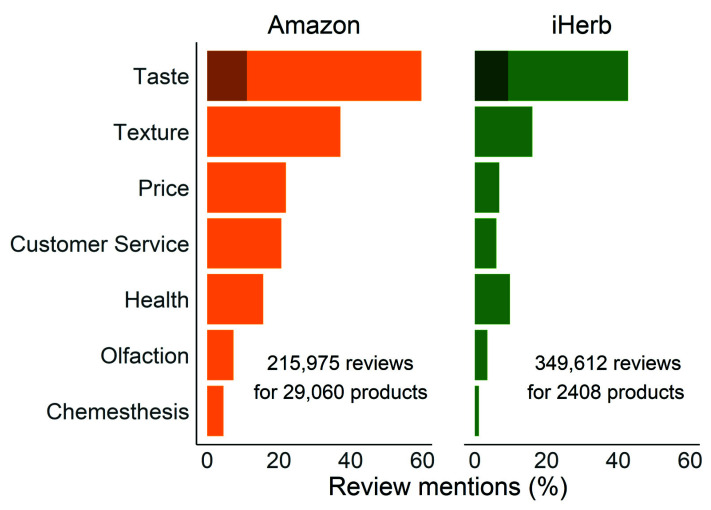
Percentage of reviews containing words from each category in the Amazon and iHerb datasets. The darker bars in the “taste” category indicate the percentage of reviews with the word “sweet”.

We collected over 200,000 reviews of ~30,000 food products on Amazon and ~350,000 reviews of ~2400 food products on iHerb. The Amazon reviews set was previously analyzed by Reed et al. [27]. Here, we scraped additional product information from Amazon.com. The iHerb dataset was created by scraping products and reviews under the “grocery” category of iHerb.com. We found that “taste” and “texture” related words were the most common in both datasets (Figure 1). Following those, price and customer service are mentioned with the same frequency on Amazon, followed by health (22%, 22%, and 14%, respectively), while in iHerb, words relating to health (10%) are more common than those relating to price (7%) and customer service (6%). Sweetness is the most frequently mentioned taste modality (13.32% in Amazon and 9.41% in iHerb) and is more common than words relating to olfaction and chemesthesis in both datasets, and more than price and customer service in the iHerb dataset (the words used in the search are detailed in Section 2.2).

Tallying mentions of different sweetness levels was first performed, as described in [27], returning the same results (Supplementary Table S1). Similar results were also obtained for the subset of Amazon reviews used here (a detailed description of the dataset is provided in Section 2.1), with deviations of 0.6%, 0%, and −0.6% for oversweet, under-sweet, and neutral levels, respectively (Table 1 “Reed’s phrases”). 

Next, extended sets of phrases, as described in Section 2.3, were used. In brief, we added more oversweet phrases, as well as more negations to these phrases. This increased the neutral and under-sweet phrases (see List S1 in the Supplementary Information for the full list of phrases). The numbers of phrases for each sweetness level are presented in Table 2.

Since mentioning the word “sweet” without the connotation of over or under sweetness can be inferred as neutral, the total mentions of neutral sweetness are the most common, with 66% and 65% occurrences out of all mentions of sweetness in Amazon and iHerb, respectively (Table 1, “This paper’s phrases”).

Mentions of sweetness level in both datasets are similar and exhibit similar trends. Adding further phrases and negations cuts the mentions of oversweetness by half (from 56.8% and 52.4% to 22.6% and 27.5% in Amazon and iHerb, respectively).

In both datasets, the most common mention of sweetness is neutral (65.6% in Amazon and 64.5% in iHerb), followed by oversweetness (22.6% in Amazon and 27.5% in iHerb). “Under-sweet” counts went up from 186 and 447 to 3726 and 2308 for Amazon and iHerb, respectively, following the use of the additional phrases. Mentions of oversweetness are only 1.9 and 3.4 times as common as mentions of under-sweetness, a much lower difference than previously stated [27]. These significant differences emphasize that comprehensive linguistic approaches are essential in order not to miss relevant mentions and, most crucially, to eliminate irrelevant ones.

Regarding the reviews containing oversweet phrases, one can easily see that some do not necessarily mean the reviewer perceives the product as oversweet; for example, the following Amazon reviews: “I have had waffles from other mixes that are too sweet. I have also made them from scratch. I like my waffles with no sugar in them because I don’t like too much sugar. This mix is very good. Waffles are just the right consistency for me and the mix is easy to prepare.” by customer A274UPTASJHAL4 for product B001E5E29A; “I have tried many of the no sugar sweeteners out there. I find this to be the best. I like it even more than truvia (which is way too sweet for me). I put it in everything where I used to put sugar. Love it. Good stuff.” by customer A2GEZJHBV92EVR for product B00374ZKQ0.

These examples emphasized the need to classify all the reviews that contain oversweet phrases. The Amazon reviews were classified manually (*n* = 7126). For iHerb, 5590 reviews were classified manually, and then were used to develop an XGBoost classifier (see Methods), which was used to classify the remaining reviews (*n* = 2273).

The classifier achieved accuracies of 81 ± 1%, 79%, and 84% on the training set (averaged), test set, and holdout set, respectively. The percentages of remaining oversweet reviews out of those classified were similar—57% (3179/5590) and 63% (1426/2273) for the reviews classified manually and using the classifier, respectively.

After this additional classification, 7.1% (*n* = 2242, Amazon) and 16.1% (*n* = 4605, iHerb) of the products remained labeled as oversweet out of the total mentions of sweetness. 

Thus, we can summarize that the more careful the analysis of the reviews, the fewer occurrences of oversweetness relating to the reviewed products remain. Nevertheless, oversweetness is still non-negligible. We next assessed the effect of oversweetness on customer satisfaction. 

Specifically, we set out to examine whether “oversweet” mentions are linked to diminished product liking. All products with over 50 reviews and at least 10% “oversweet” reviews from both sources (10 in Amazon, 18 in iHerb) were analyzed. Some of the iHerb reviews were deleted from iHerb.com between the initial scraping and the time of analysis (last accessed on 22 June 2022), which resulted in the elimination of one of the products by said criteria, leaving 26 products in total.

**Figure 2:**
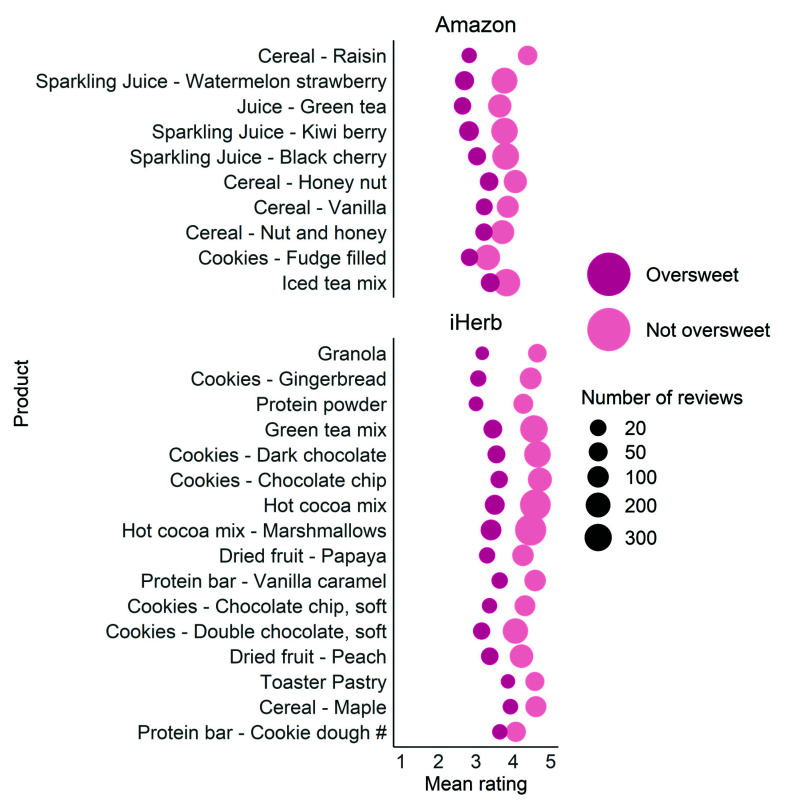
Mean ratings for oversweet and not oversweet reviews for each product. Dot sizes indicate the number of reviews for each group and product. # indicates that difference in average ranking is not significant.

The average ratings of all oversweet reviews and the rest of the reviews for each of the 26 products were calculated and compared. For 25 products, the mean ratings of the oversweet reviews were significantly lower than the not oversweet reviews (Figure 2).

This result suggests that for some products, at least 10% of the consumers find it oversweet and rate it significantly lower than those who do not perceive it as oversweet. One could infer that these customers would prefer a less sweet version of the product. 

**Figure 3:**
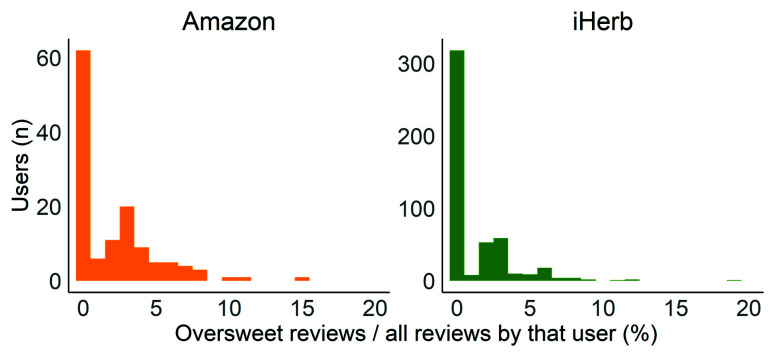
Oversweet review frequency. Frequency of oversweet reviews out of the total reviews posted by each customer for customers who posted over 30 reviews.

To establish whether the same group of people generally tend to review products as oversweet, we tallied the number of customers who posted over 30 reviews. The average ratio of oversweet reviews per reviewer is 2.1 ± 2.7 for Amazon customers and 1.3 ± 2.2 for iHerb customers. The vast majority of customers never complained about oversweetness, and only 3/128 Amazon customers and 4/492 iHerb customers submitted over 10% oversweet reviews. This indicates that oversweetness perception is not due to a few consumers complaining about multiple products but is rather derived from a non-negligible number of customers reacting in a product-dependent manner (Figure 3).

Interestingly, all but two products in Figure 2 contain caloric sweeteners, with eighteen of them containing sucrose. We next set out to find which sweeteners tend to appear in more “oversweet” reviews than others, analyzing all 215,975 reviews from Amazon and 349,612 reviews from iHerb.

**Figure 4:**
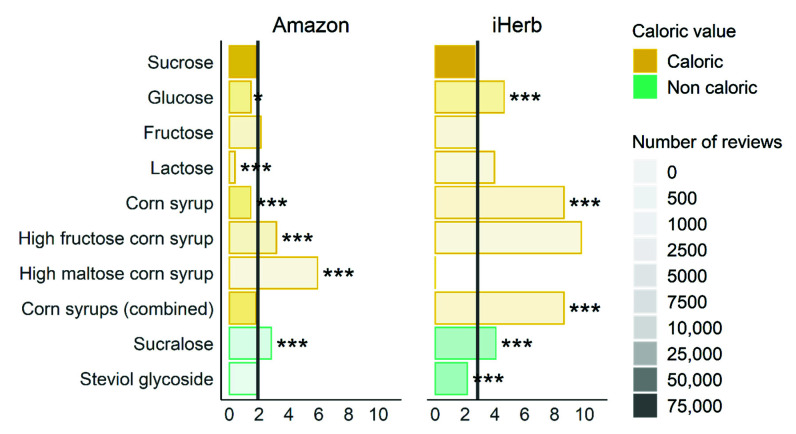
Oversweet reviews out of total reviews of products containing each sweetener. Only the most frequently used sweeteners are displayed. The transparency level indicates the number of reviews; the color indicates sweetener type; vertical lines represent the average percentage (%) of oversweet reviews; * *p* < 0.05, *** *p* < 0.001.

The frequency of “oversweet” reviews out of all reviews is 1.9% on Amazon and 2.8% on iHerb, consistent with 7.1% (Amazon) and 16.1% (iHerb) of all reviews labeled as oversweet. This is partially driven by sucrose which is used in 37% (10,640/29,060) of the Amazon products and 28% (663/2408) of the iHerb products, and has an oversweet review ratio of 1.8% in Amazon and of 2.7% in iHerb,3 as seen in Figure 4. 

The proportion of oversweet reviews out of total reviews containing each sweetener against the general proportion of oversweet reviews in the dataset highlights the most frequently used sweeteners in Figure 4. (see Figure S1 in the Supplementary Information for a full figure containing all examined sweeteners).

Sucrose was the most common sweetener and established the average percentage of oversweet reviews for all products. The average oversweetness of sucrose-containing products was higher in iHerb. Compared to the average percentage of oversweet reviews (1.9% on Amazon and 2.8% on iHerb), glucose was less frequently found in products with oversweet reviews on Amazon but more frequent than others on iHerb. 

Reviews of products with lactose had few mentions of oversweetness in Amazon (0.4%) but more in iHerb (4.0%). This is interesting in view of the lower sweetness of lactose compared to other sugars [37]. Corn syrups had a particularly high representation of oversweet reviews in iHerb. 

Sucralose had a consistent and significant tendency for oversweetness in both datasets, while steviol glycoside was not more frequently oversweet than others. This might be due to its bitterness or aftertaste, especially at higher concentrations [38,39], which are consequently avoided during product development. The frequent use of sucralose in iHerb (5.6% of the products as opposed to 2.3% of the Amazon products) is an additional reason for more oversweetness cases.

When grouping sweeteners by caloric value (caloric, non-caloric, sugar alcohol, sugar fiber), we found that in the Amazon dataset, non-caloric sweeteners were significantly more likely to be classified as oversweet, while in iHerb caloric sweeteners were significantly more likely not to be classified as oversweet (see Supplementary Figure S2 for details).

Parameters that made customers prefer one sweetener over another include concentration, aftertaste, different number of sachets needed for a cup of coffee, consistency, and delivery method (powder, crystals, or liquid). Furthermore, some people reported needing to use just half a sachet for a cup of coffee, while others reported using a few sachets.

## 4. Discussion

Due to the innate attraction to the sweet taste, there is tension between the attempt to produce healthier foods and the incentive to make them palatable and attractive to the consumers.

Following [27], we investigated online reviews of food products that were reported to be too sweet. Oversweetness is the most commented concern regarding taste, thus providing the potential to improve product taste and healthiness simultaneously. 

### 4.1. The Importance of Review Interpretations

We added 275 more phrases than previously examined by Reed et al. [27] to better reflect the mentions of sweetness. We demonstrated that using more phrases, in addition to the general “sweet” only category to reflect additional neutral mentions of sweetness, reduces the significance of oversweetness out of the total mentions of sweetness. In addition, classifying the reviews to indicate the true meaning of oversweetness indicated that oversweetness is not as concerning a problem, as previously mentioned. In addition, it allowed us to connect the oversweetness perception to specific products and, by extension, to their ingredients. While previous research examined sentiment in reviews and its correlation to score and helpfulness [40,41], no research was conducted to classify the specific attributes in the reviews and their connection to the ingredients.

### 4.2. Amazon vs. iHerb 

The importance of taste is in line with numerous studies [27,42,43]. However, a study performed on ~1000 participants from three Chinese cities exploring the food choice in different modes of e-commerce shopping [44], found that taste had a significant effect on food choice in various modes of e-commerce food services, e.g., takeaway delivery apps but surprisingly not in business-to-consumer e-commerce platforms, such as Amazon.com. This inconsistency with our results can be explained by our use of significantly more participants and cultural backgrounds.

Contrary to Amazon, iHerb customers mention health relatively more than price and customer service. This aligns with iHerb’s mission statement of being the first choice for health and wellness (https://www.iherb.com/info/about, accessed on 22 June 2022). On the other hand, Amazon’s slogan of “Spend less. Smile more”. This indicates a match between the companies and their target audiences’ expectations of the products sold on each website. While we found that in both datasets, sweetness is the most frequently mentioned taste modality, similarly to [27], oversweetness referring to the products themselves is much more common in the iHerb reviews (16%) than the Amazon reviews (7%). 

### 4.3. Linking Reviews to Ingredients

Products containing sucralose tended to be perceived as oversweet in both datasets compared to other sweeteners. Sucralose is a non-caloric sweetener about 600 times as potent as sucrose [45,46]. Since this tendency is consistent in both datasets, we assume that there is a considerable fraction of the population that would benefit from a line of products with lower levels of sucralose.

Some sweeteners had significant tendencies for oversweetness in just one of the datasets, which can be explained by their different prevalence in the different datasets, affecting the statistical significance (e.g., high fructose corn syrup is in 3.7% of Amazon products (*n* = 1064) but only in 0.12% iHerb products (*n* = 3)). 

Sucrose is the most common sweetener in both datasets, and sucrose-containing products are found to be oversweet more frequently by iHerb customers.

### 4.4. Sweetness Intensity Preference

Sweetness intensity preference varies from person to person and depends on numerous biological and environmental parameters [1,5,47], and liking sweet taste can depend on genetics [48]. Generally, sweet-taste-liking response is characterized as either positive, with an increase of sweetness intensity, negative, or an inverted-U shape [49], with no consensus on the optimal way to identify these phenotypes [50]. Overall, people who prefer lower sweetness intensity are likely to rate highly sweet products lower than those with a higher sweetness intensity preference [20].

For these reasons, we expected customers who reported on oversweetness to generally perceive products as too sweet and to, thus, have a much higher portion of oversweet comments out of their overall reviews. Surprisingly, we found that the oversweet reviews are distributed over multiple customers, while the same customers complain about the oversweetness of some products but not of others, indicating the important effect of the product itself. 

On average, people who reviewed products as oversweet tended to award them significantly lower rating scores. This implies that, at least in some cases, oversweetness is an unwanted quality that damages consumers’ satisfaction with products and should be addressed to increase positive responses. For these products, introducing a less sweet version in addition to the classical one, will benefit the consumers by providing a healthier and tastier option and the company by increasing the number of satisfied customers.

### 4.5. Potential Marketing Strategies

Food companies are aiming to reach the optimal sweetness and maximal palatability of their products. Yet, even sweets, such as cookies and sparkling juices, are too sweet for some consumers. For example, 24% (65/340) of consumers found the kiwi berry juice oversweet and gave it a rating lower by 0.9 stars on average than those who did not find the product too sweet. Approximately 16% (29/213) of consumers found the chocolate chip cookies oversweet with a score difference of 1.1 stars (Figure 2). With a carbonated soft drinks market of USD 222 billion [51] and USD 31 billion for cookies in 2018 [52], even if a small percentage of the consumers prefer a less sweet version of these products, there is a large market for slightly less sweet (and healthier) versions of the products. Adding the option to purchase less sweet versions of existing products is a venue that may prove beneficial to both companies and consumers.

### 4.6. Limitations and Insights for Future Directions

Customer reviews provide a valuable source of spontaneous responses. They are considered trustworthy and are used as a marketing tool by retailers to promote new products [53,54]. Researchers use these reviews to study a wide variety of subjects, including identifying product aspects and customer focus [55,56]; examining consumer emotion, satisfaction, and general experience [56,57]; and measuring the impact of visual information on behavior [58]. However, those who write reviews online (a) shop for groceries online and (b) either enjoy writing reviews or wish to receive rewards as part of a rewards program. This population might not accurately represent the majority of consumers, excluding, for example, young children, the elderly, and people who do not wish to write reviews, and these caveats should be kept in mind. Furthermore, the reviews studied here are in English and, therefore, represent English-speaking consumers. Future research can make use of the different languages used in these websites and, of course, study other popular online stores. By doing so, they will provide insights into the different cultures and target audiences. 

Regarding the classification of oversweetness perception, we used a binary scale to find all oversweet reviews. A more fine-grained scale gradually ranging from under-sweetness to oversweetness could be used instead.

While we focused on taste only, other parameters influence sweetness perception, such as odors [59,60], degree of processing [61], texture [62], and visual perception [63]. Future research can integrate these parameters to better understand their influence. Moreover, more variables can be examined for their influence on taste perception, including combinations of different sweeteners and their synergistic effects [46,64], as well as the suppressive or masking effects of other substances [65].

## 5. Conclusions

Using big data to analyze taste perception allows achieving vast information in a fairly quick and cost-effective way. This provides an additional tool for monitoring the acceptance of food products after they enter the market.

We found that a non-negligible proportion of online food product reviews mentioning sweetness consider them oversweet (7% Amazon, 16% iHerb), and that certain sweeteners are more likely to cause oversweetness than others. The tension between taste and health is a constant challenge for the modern food industry and the consumers. Here, we show that some “extra-sweet” products are an overshoot for some consumers, resulting in a misfit, both in terms of hedonics and in nutritional parameters.

Considering that consumers who perceive products as oversweet award them with lower scores and that phrases relating to products not being sweet enough were rare, we recommend paying special attention to the potential of providing lower sweetness-level versions of some products. Particular attention should be paid to products sweetened with sucralose or corn syrups.

## Figures and Tables

**Table 1:** Overall mentions of sweetness by sweetness level.

	Reed’s Phrases				This Paper’s Phrases				After Classification			
	Amazon (215,975 Reviews)		iHerb (349,612 Reviews)		Amazon (215,975 Reviews)		iHerb (349,612 Reviews)		Amazon (215,975 Reviews)		iHerb (349,612 Reviews)	
	Count	%	Count	%	Count	%	Count	%	Count	%	Count	%
Oversweet	5008	56.8	6187	52.4	7126	22.6	7863	27.5	2242	7.1	4605	16.1
Under-sweet	186	2.1	447	3.8	3726	11.8	2308	8.0	29,294	92.9	24,022	83.9
Neutral	3629	41.1	5162	43.8	3562	11.3	6383	22.3				
“Sweet” only	Not checked				17,122	54.3	12,073	42.2				

**Table 2:** Numbers of phrases used to evaluate sweetness in Reed et al. [27] and in this paper. “Sweet” only refers to the mention of the word “sweet” that is not an occurrence of one of the phrases categorized specifically as over, under, or neutral in this table.

	Reed’s	This Paper
Oversweet	16	139
Under-sweet	3	50
Neutral	19	124
“Sweet” only	Not checked	1

## Data Availability

The data presented in this study are available on request from the corresponding author.

## Supplementary Materials

The following supporting information can be downloaded at: https://www.mdpi.com/article/10.3390/foods11131872/s1, List S1: Phrases used for levels of sweetness with at least one occurrence in the dataset; Table S1: Overall mentions of sweetness by sweetness level according to Reed et al. (2019); Figure S1: Oversweet reviews out of all reviews of products containing each sweetener; Figure S2: Oversweet reviews out of all reviews of products containing each sweetener category.
