# Supplementary material for: Harnessing Food Product Reviews for Personalizing Sweetness Levels
Source: Foods. 2022 Jun 24;11(13):1872. doi: 10.3390/foods11131872 (PMC9266276; doi:10.3390/foods11131872)
Supplement: Supplementary file 1 [file foods-11-01872-s001.zip › foods-1770383-supplementary.pdf]

Supplementary Data

List S1: Phrases used for levels of sweetness with at least one occurrence in the dataset:

Oversweet:

absolutely sweet, achingly sweet, actual sweet, actually sweet, aggressive sweetness, alittle sweet, annoyingly sweet, awfully sweet, bit sweet, cloying sweet, cloyingly sweet, considerably sweeter, crazy sweet, deadly sweet, definitely sweet, definitely sweeter, disgusting sweet, disgustingly sweet, especially sweet, even sweeter, exceedingly sweet, excess sweetness, excessive sweetness, excessively sweet, extra sweet, extraordinarily sweet, extreamly sweet, extreme sweetness, extremely sweet, extremly sweet, fairly sweet, far sweeter, feels sweeter, harsh sweetness, heavily sweet, heavy sweet, high sweet, high sweetness, highly sweet, horrible sweetness, horribly sweet, if not too sweet, insanely sweet, intense sweet, intensely sweet, it's sweeter, kinda sweet, light sweet, lightly sweet, lot sweet, more sweet, mostly sweet, much sweet, nasty sweet, nauseatingly sweet, noticeably sweet, oddly sweet, on a sweet side, on the sweet side, ooooooo sweet, over sweet, over the top sweet, overall sweet, overally sweet, overbearingly sweet, overly sweet, overpowering sweet, overpoweringly sweet, oversweet, overt sweetness, overtly sweet, overwhelming sweet, overwhelmingly sweet, painfully sweet, particularly sweet, plenty sweet, potent sweetness, powerful sweet, poweringly sweet, pretty sweet, quiet sweet, quite sweet, rather sweet, real sweet, really sweet, reasonably sweet, relatively sweet, ridiculously sweet, sickening sweet, sickeningly sweet, sickenly sweet, sickly sweet, sicky sweet, significantly sweeter, slighly sweet, slightest sweet, slighty sweet, so sweet, sooo sweet, soooo sweet, sooooo sweet, sticky sweet, strangely sweet, strong sweet, strongly sweetened, sugary sweet, super duper sweet, super sweet, suprisingly sweet, surprisingly sweet, sweet sugary, syrupy sweet, tad sweet, terribly sweet, the sweetest, to sweet, too much sugar, too sweet, tooo sweet, toooo sweet, tooooo sweet, toooooo sweet, tooooooooo sweet, totally sweet, uber sweet, ultra sweet, unbearably sweet, unbelievably sweet, unnaturally sweet, unnecessarily sweet, unnecessary sweetness, unpleasant sweet, unpleasant sweetness, unpleasantly sweet, unusually sweet, v sweet, verry sweet, very sweet, weirdly sweet

Under-sweet:

almost sweet, almost sweet enough, aren't sweet, aren't sweet enough, barely sweet, hardly sweet, isn't sweet, isn't sweet enough, isnt sweet, lacking in sweet, lacks sweetness, least sweet, less sweet, less sweet enough, minimal sweetness, minimally sweet, need sweet, needs sweet, no sweet, nor sweet, nor sweet enough, not actually sweet, not as sweet, not enough sweet, not exactly sweet, not exceptionally sweet, not much sweet, not nearly sweet enough, not noticeably sweet, not particularly sweet, not quite sweet, not quite sweet enough, not real sweet, not reali sweet, not really sweet, not really sweet enough, not so sweet, not sugary sweet, not sweet, not sweet enough, nothing sweet, wasn't sweet, wasn't sweet enough, wasn`t sweet enough, wasnt sweet, wasnt sweet enough, weren't sweet, weren't sweet enough, without being sweet, and without sweet

Neutral:

absolutely sweet enough, already sweet, already sweet enough, always sweet, always sweet enough, amazingly sweet, as sweet, as sweet enough, awesome sweet, balanced sweet, best sweetest, definitely sweet enough, delicate sweetness, delicately sweet, delicious sweet, deliciously sweet, delightful sweet, delightfully sweet, enough sweet, ever so slightly sweet, exceptionally sweet, fabulous sweet, faint sweet, faintly sweet, fantastic sweet, gentle sweet, good sweet, great sweet, incredible sweet, incredibly sweet, lightly sweet enough, little sweet, lovely sweet, lusciously sweet, medium sweet, mild sweet, mild sweet enough, mildly sweet, moderate sweet, moderately sweet, nice sweet, nicely sweet, not cloying sweet, not cloyingly sweet, not crazy sweet, not deadly sweet, not disgustingly sweet, not exceedingly sweet, not excessive sweetness, not excessively sweet, not extra sweet, not extremely sweet, not good sweet, not great sweet, not heavily sweet, not horribly sweet, not incredibly sweet, not insanely sweet, not intense sweet, not obnoxiously sweet, not on the sweet side, not over sweet, not over the top sweet, not overally sweet, not overbearingly sweet, not overly sweet, not overpowering sweet, not overpoweringly sweet, not oversweet, not overtly sweet, not overwhelming sweet, not overwhelmingly sweet, not ridiculously sweet, not sickening sweet, not sickeningly sweet, not sickenly sweet, not sickly sweet, not sicky sweet, not sticky sweet, not strong sweet, not strongly sweetened, not super sweet, not syrupy sweet, not terribly sweet, not the sweetest, not to sweet, not too much sugar, not too sweet, not tooo sweet, not toooo sweet, not tooooo sweet, not tooth achingly sweet, not totally sweet, not uber sweet, not ultra sweet, not unnaturally sweet, not unnecessary sweetness, not very sweet, not weirdly sweet, not wicked sweet, perfect sweetness, perfectly sweet, perfectly sweet enough, pleasant sweet, pleasantly sweet, pleasingly sweet, plenty sweet enough, quite sweet enough, really sweet enough, right sweet, satisfying sweetness, satisfyingly sweet, satisfyingly sweet enough, scrumptious sweet, so sweet enough, subtle sweet, sufficient sweetness, sufficiently sweet, sweet as i would prefer, sweet enough, sweet enough enough, sweetened enough, wonderful sweetness, yummy sweet

Table S1: Overall mentions of sweetness by sweetness level according to Reed et al. (2019). Replication of the phrase search done by (Reed et al., 2019), to accompany Table 2. Our results stand in full accordance with Reed's.

	Reed's phrases, original dataset (393,598 reviews)	
	Count	%
Oversweet	7,230	56.2
Under-sweet	268	2.1
Neutral	5,370	41.7
Just "sweet"	Not checked	

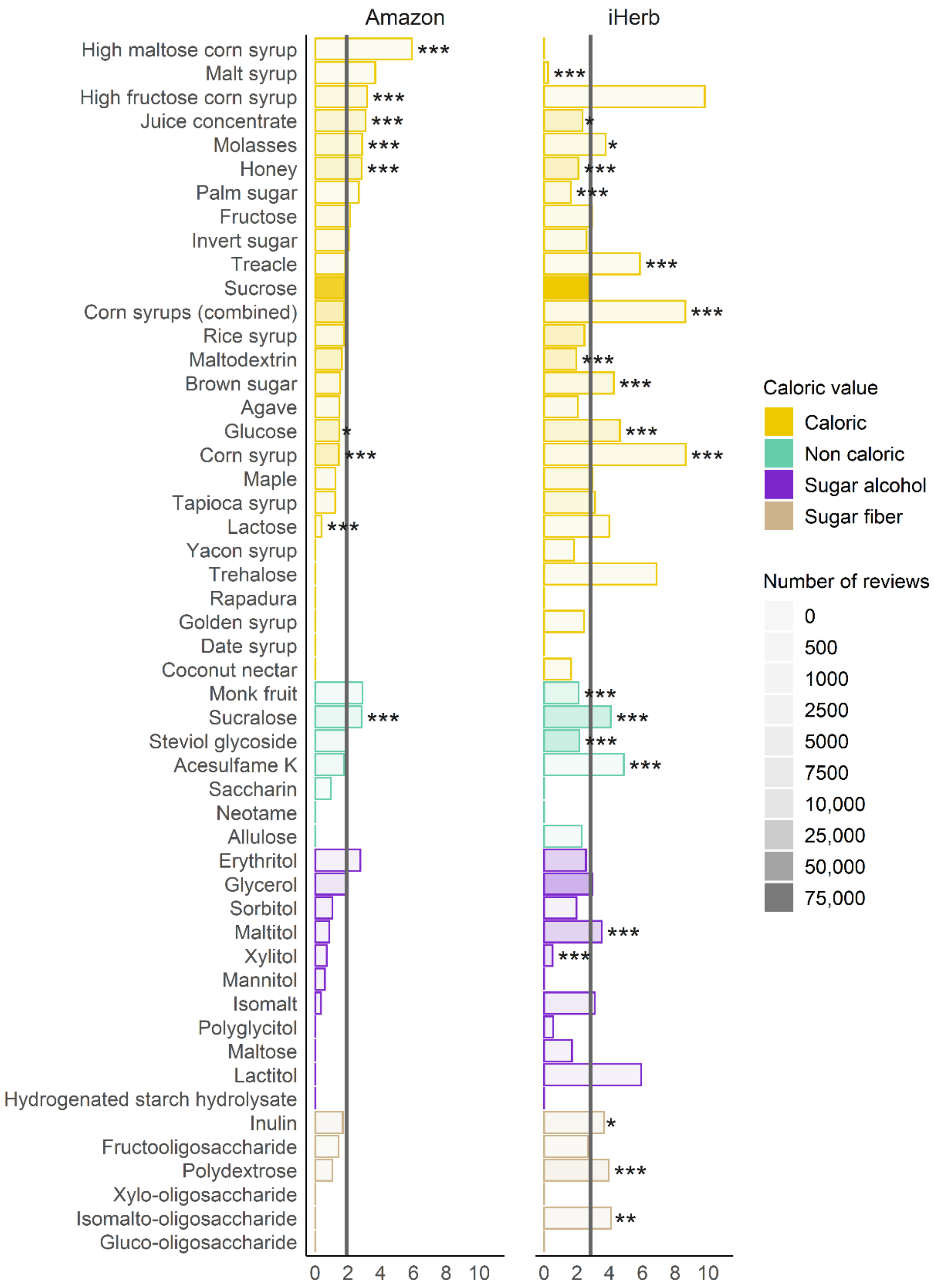

Figure S1: Oversweet reviews out of all reviews of products containing each sweetener. Transparency indicates number of reviews; color indicates type of sweetener; vertical lines represent the average % of oversweet reviews.

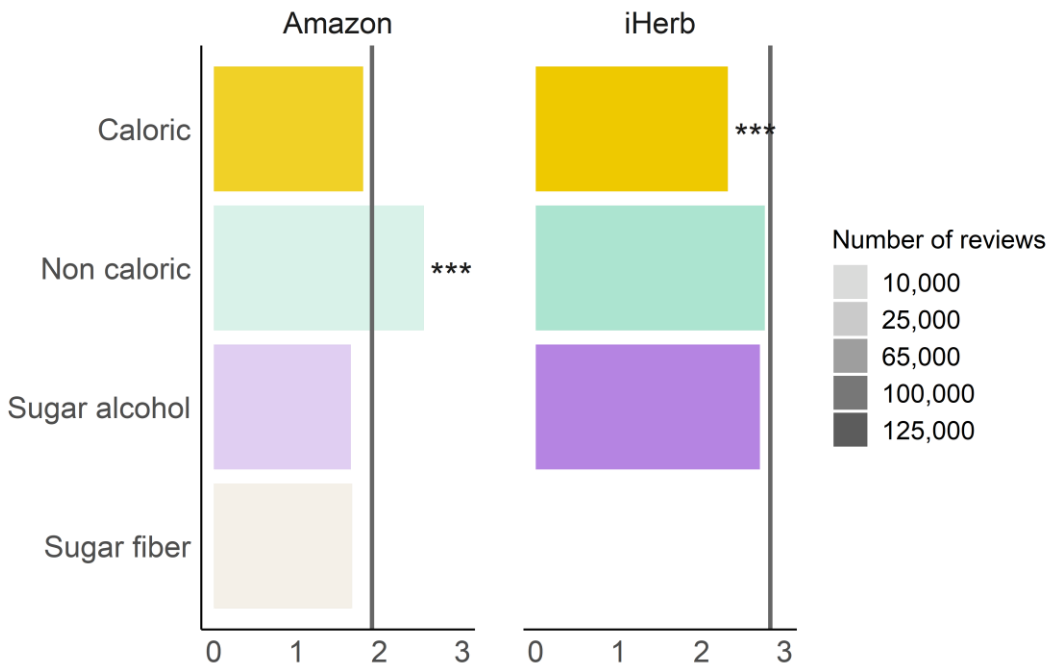

Figure S2: Oversweet reviews out of all reviews of products containing each sweetener type. Transparency indicates number of reviews; vertical lines represent the average % of oversweet reviews.
